# Anti-Cariogenic Effect of Trans-Cinnamaldehyde in an In Vitro Mouse Jaw Explant Model

**DOI:** 10.3390/ph19040566

**Published:** 2026-04-01

**Authors:** Zilefac Brian Ngokwe, Amit Wolfoviz-Zilberman, Galia Blum, Talya Hanna Avraham, Nurit Beyth, Yael Houri-Haddad, Dana Kesler-Shvero

**Affiliations:** 1 Department of Prosthodontics, Hadassah Medical Center, Faculty of Dental Medicine, Hebrew University of Jerusalem, Jerusalem 9112001, Israel; brian.ngokwe@mail.huji.ac.il (Z.B.N.); nurit.beyth@mail.huji.ac.il (N.B.); yaelho@ekmd.huji.ac.il (Y.H.-H.); dana.kesler@mail.huji.ac.il (D.K.-S.); 2 The International Graduate Program in Biomedical Sciences, Faculty of Dental Medicine, Hebrew University of Jerusalem, Jerusalem 9112001, Israel; 3 The Institute for Drug Research, School of Pharmacy, Faculty of Medicine, Hebrew University of Jerusalem, Jerusalem 9112001, Israel; galiabl@ekmd.huji.ac.il (G.B.); talya.avraham@mail.huji.ac.il (T.H.A.)

**Keywords:** anti-cariogenic, trans-cinnamaldehyde, mouse jaw explant model, *S. mutans*

## Abstract

**Background**: Dental caries, primarily caused by *Streptococcus mutans* (*S. mutans*), is a prevalent condition with significant global impact. Trans-cinnamaldehyde (TC), a phytochemical derived from the cinnamon plant, has shown promising antibacterial and antibiofilm activity against *S. mutans*. This study aimed to evaluate the anti-cariogenic effects of TC on *S. mutans* using an innovative mouse jaw explant model. **Methods**: TC was diluted in an organic solvent across various concentrations. Initially, cytotoxicity assays were performed at all tested TC concentrations. Sub-minimum bactericidal concentrations were then used to examine the distribution and morphology of *S. mutans* biofilms. Hemi-mandibles were dissected from euthanized, healthy, seven-week-old female mice to study the impact of TC on the cariogenic activity of *S. mutans* using stereoscopic analysis. Finally, pH changes during exposure to cariogenic conditions and post-treatment bacterial viability were measured. **Results**: In vitro data demonstrate that TC doses of ≤625 µg/mL were non-cytotoxic. Treatment groups exposed to TC exhibited altered bacterial morphology, including abnormal and incomplete cell division. In the mouse jaw explant model, TC doses of ≥625 µg/mL showed anti-cariogenic effects, evidenced by the absence of visible carious lesions. Additionally, pH changes and post-treatment viable bacterial counts corresponded with the observed anti-cariogenic activity. TC doses ≤625 µg/mL led to a pH drop over time and the presence of bacterial colonies. **Conclusions**: TC exhibits significant anti-cariogenic activity against *S. mutans* in the mouse model. Our findings suggest that 625 µg/mL is the lowest non-toxic concentration of TC that effectively inhibits cariogenic activity.

## 1. Introduction

Dental caries affects about half of the global population and is one of the most widespread oral diseases, as reported in the Global Status Report on Oral Health 2022 [1]. It ranks sixth with respect to DALYs (disability-adjusted life years), underscoring its significant impact and recognition as a major global public health burden [2].

Biofilms, irreversibly attached communities of microbial cells embedded within an extracellular polymeric matrix [3], were first observed by Antonie van Leeuwenhoek on tooth surfaces [4], although evidence of biofilms dates back over 2000 years. These microbial communities possess a range of defense mechanisms, including quorum sensing for communication, and they impose substantial financial burdens across various sectors, particularly healthcare.

Oral biofilms play a central role in the development of dental caries, the most common pathology affecting the oral cavity. Microorganisms within these biofilms colonize tooth and gum surfaces, with *S. mutans* being one of the primary etiological agents involved [5,6,7,8]. They possess acidogenic and aciduric characteristics [9,10], which cause carious lesions [8,10].

Fluoride is the most widely utilized agent for the prevention of dental caries and is commonly incorporated into a range of oral care products such as toothpastes, mouthwashes, and gels [11,12]. However, certain bacteria have developed varying levels of tolerance to fluoride exposure, leading to the emergence of fluoride resistance.

*S. mutans* naturally resides in the human oral cavity, particularly within dental plaque, a multispecies biofilm that forms on the hard surfaces of teeth [13]. In our previous study, we demonstrated both antibacterial and antibiofilm activity of TC against *S. mutans*, including on preformed biofilms that closely mimic natural conditions. These experiments utilized hydroxyapatite, which is the primary inorganic component of tooth enamel, as the substrate [14,15]. The treatment involved TC, a naturally occurring phytochemical derived from the cinnamon plant, which offers several benefits, including a relatively novel chemical structure that may help evade existing bacterial resistance mechanisms [16].

Additional antibacterial strategies targeting *S. mutans* have been reported, including blue light therapy [17], phage therapy [18], and the use of cannabinoids [10,19].

In this research, we used the in vitro mouse jaw explant model [20], which enables the consistent formation of extensive carious lesions using *S. mutans* within 5 days, thus enabling a more comprehensive investigation of the anti-cariogenic effects of TC.

### Our Working Hypothesis

We have previously detailed the antibacterial and antibiofilm effects of TC against *S. mutans* [15]. We aimed to investigate the potential anti-cariogenic activity of TC using a jaw explant model. This model was deliberately chosen as it helps minimize unnecessary animal sacrifice. We hypothesize that TC will exhibit dose-dependent anti-cariogenic effects on mouse teeth using the described in vitro model.

## 2. Results

### 2.1. Cytotoxicity Assay

**Figure 1:**
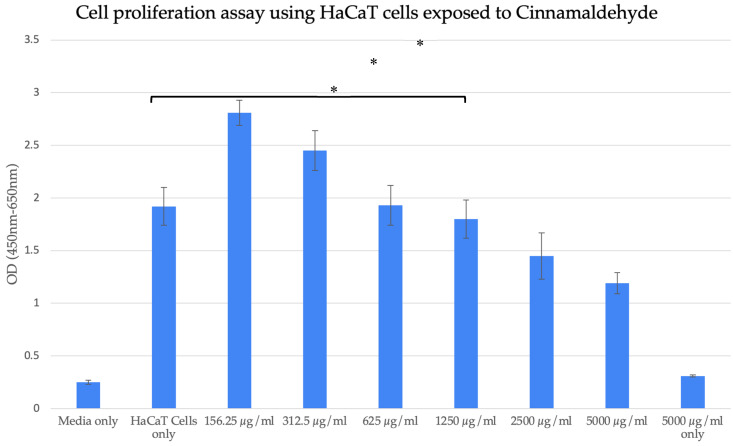
Cell proliferation assay (toxicity). TC doses ≤ 625 µg/mL were not cytotoxic. * *p* < 0.05 (8 wells per concentration, three repetitions). (The samples were compared to the HaCaT cells only group, which served as a reference, and is the 5000 µg/mL only group seen in Figure 1).

We performed a cytotoxicity assay using HaCaT cells and observed that TC concentrations up to 625 µg/mL were non-cytotoxic. Additionally, TC density did not influence the results, as the highest concentration tested (5000 µg/mL) exhibited a density comparable to that of the culture media alone, as illustrated in Figure 1.

### 2.2. High-Resolution Scanning Electron Microscopy (HR-SEM)

**Figure 2:**
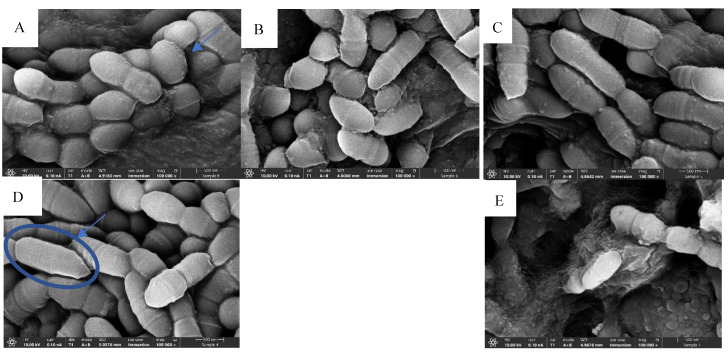
HR SEM images of *S. mutans* following contact with different TC concentrations at 100,000 magnifications. Biofilm was grown on HA discs for 24 h using different TC concentrations at 100,000 magnifications: (**A**) untreated *S. mutans* control; (**B**) 156.25 µg/mL; (**C**) 312.5 µg/mL; (**D**) 625 µg/mL; (**E**) chlorhexidine control.

Bacteria in the treatment groups exhibited morphological changes, notably abnormal cell division. As shown in Figure 2B–E, the bacterial cells appear relatively elongated, indicating incomplete cell division, in contrast to the untreated group shown in Figure 2A. These differences are further highlighted in Figure 2A–D; as the TC concentrations increased, syncytium-like cells can be observed.

Normal morphology and cell division are shown in the untreated group (Figure 2A); morphological changes and syncytium-like cells (blue circle) can be observed as the TC concentration rises (Figure 2D).

### 2.3. Mouse Jaw In Vitro Caries Model

#### 2.3.1. Clinical Evaluation

**Figure 3:**
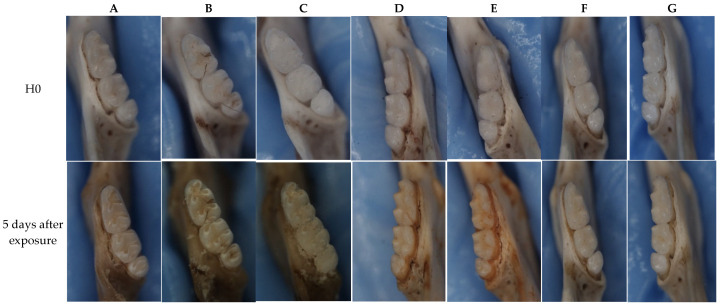
Clinical evaluation of carious lesions. Hemi-mandibles were photographed using a stereomicroscope, and the anti-cariogenic activity was evaluated before treatment and after 5 days of treatment with 5% sucrose and different concentrations of TC. All samples were treated with *S. mutans* except G. (**A**) Untreated *S. mutans* control (OD = 0.1), (**B**) 156.25 µg/mL TC, (**C**) 312.5 µg/mL TC, (**D**) 625 µg/mL TC, (**E**) 1250 µg/mL TC, (**F**) chlorhexidine (positive control), and (**G**) clear control (negative control). No visible carious lesions were observed following treatment with TC doses of ≥625 µg/mL (12 samples per concentration, three repetitions).

Using stereoscopic images, we evaluated these explants pre-treatment and 5 days after treatment with our different TC concentrations. We observed no visible caries with explants treated with TC doses ≥ 625 µg/mL, as can be seen in Figure 3. TC concentrations ranging from 625 to 5000 µg/mL exhibited a bactericidal effect, and the teeth showed no visible signs of caries. In Figure 3A–C (TC doses < 625 µg/mL), carious lesions are visible, whereas in Figure 3D–F, no signs of caries are observed.

#### 2.3.2. Quantification of Demineralization

**Figure 4:**
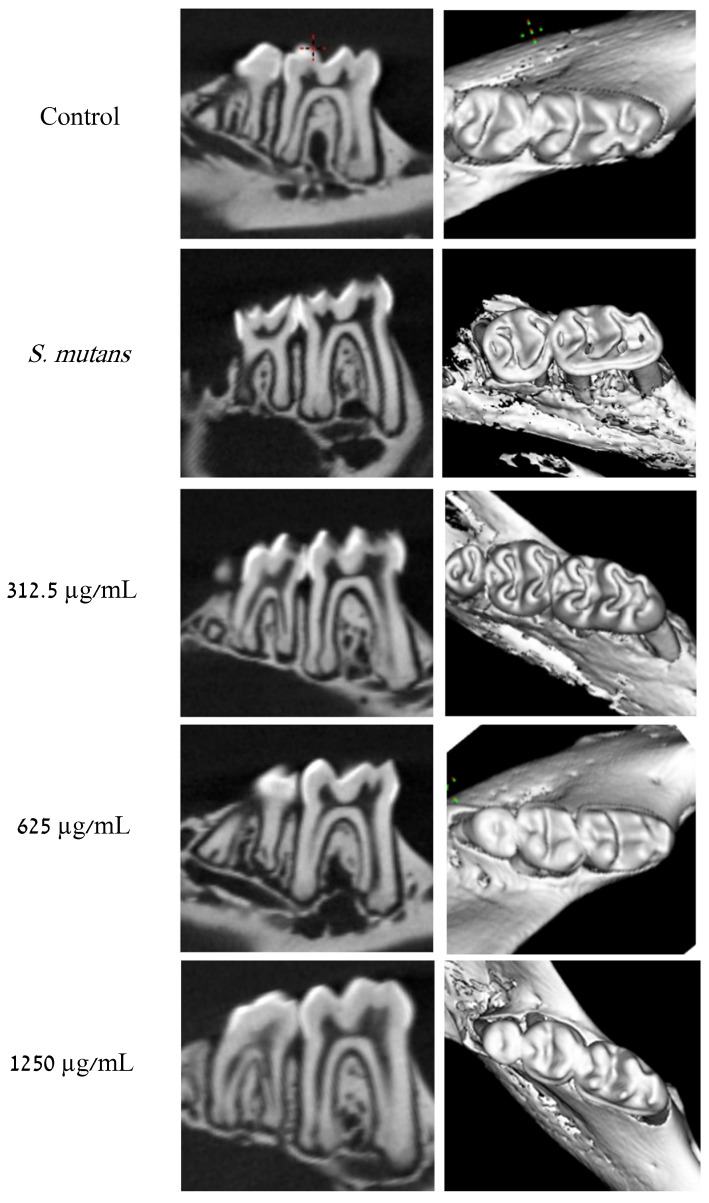
Radiographic evaluation of carious lesions. Representative radiographic image after the caries induction period. Hemi-mandibles were segmented and reconstructed to acquire 3D images by µCT. In the *S. mutans* and 312.5 µg/mL TC, we observed an important demineralization visible on the 2D coronal plane and the 3D reconstituted view. Conversely, the clear control, 625 µg/mL and 1250 µg/mL TC, showed no demineralization in both the coronal plane and the reconstituted 3D view.

Consistent with the clinical findings presented above, µCT scans (Figure 4) revealed demineralization of dental hard tissues (enamel and dentin) under caries-promoting conditions and at the suboptimal TC dose of 312.5 µg/mL. In contrast, the clear control group (without *S. mutans*) exhibited no demineralization of the hard dental tissues, a pattern that was similarly observed at the higher, suboptimal TC concentration of 625 µg/mL.

### 2.4. pH Changes over Time

**Figure 5:**
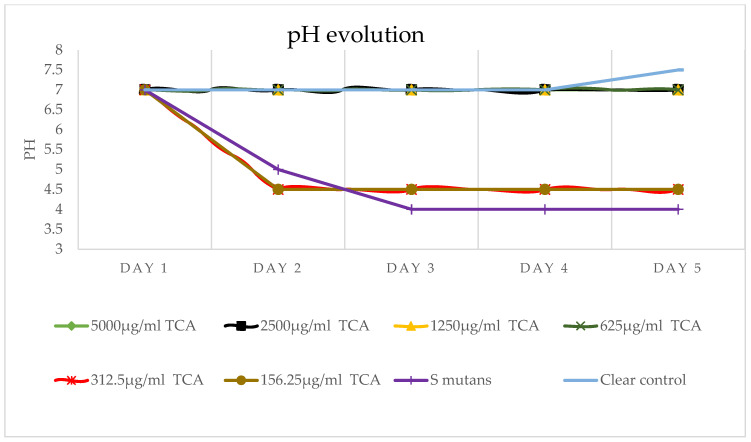
pH changes. The pH was maintained over 5 days with 625 µg/mL, maintaining a stable pH at 7 above the critical pH (experiment was repeated 5 times, *n* = 10). We have eight groups in Figure 5; on day 2, five groups, the clear control and the concentrations of 5000 µg/mL, 2500 µg/mL, 1250 µg/mL, and 625 µg/mL, are measured at pH 7. The 312.5 µg/mL and the 156.35 µg/mL concentrations are measured at pH 4.5. Because groups with the same pH substantially overlap in the figure, they can be more easily distinguished by the legend marker shape rather than the legend color.

TC concentrations of ≥625 µg/mL maintained a stable pH of around 7 throughout the 5-day treatment period, remaining above the critical threshold of 5.5 required for caries development. In contrast, the pH in groups treated with ≤625 µg/mL and in the untreated control progressively declined over time, as illustrated in Figure 5.

### 2.5. Post-Treatment Viable Counts (CFU/mL)

**Figure 6:**
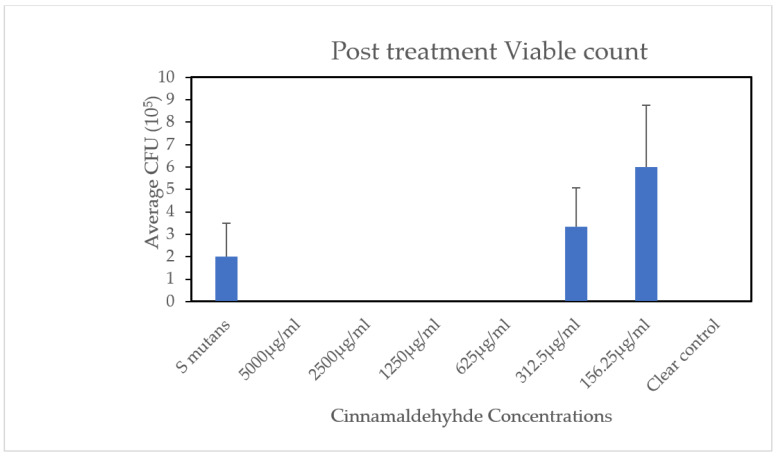
Viable bacterial counts. Post-treatment CFU counts show no colonies formed with TC doses ≥ 625 µg/mL (experiment was repeated three times, *n* = 9).

After the 5-day anti-cariogenic assessment using various TC concentrations, samples were collected from the third molars of each jaw explant using paper points to determine CFU/mL. The results, shown in Figure 6, indicate that TC doses ≥ 625 µg/mL resulted in no visible bacterial colonies. Among the groups with detectable colonies, the 156.25 µg/mL concentration showed a higher CFU count compared to the 312.5 µg/mL dose, aligning with the lower antibiofilm activity observed at 156.25 µg/mL.

## 3. Discussion

The prevention of *S. mutans* biofilm formation is a critical strategy in combating dental caries. Given the multifactorial nature of caries development, simply demonstrating the antibacterial and antibiofilm effects of TC is not sufficient. Therefore, we extended our investigation to assess the anti-cariogenic potential of TC uner controlled, caries-promoting conditions using a mouse jaw explant model. This model was intentionally selected to reduce unnecessary animal use while providing a reliable platform for evaluating caries prevention strategies.

Biofilm formation plays a central role in the initiation and progression of carious lesions. Although biofilms are found in a variety of environments, they were first described in the oral cavity. Their impact is significant, with an estimated financial burden of approximately $400 million annually on the U.S. Navy alone, underscoring the broader implications of biofilm-associated infections.

In earlier work, we characterized the antibacterial and antibiofilm activity of TC against *S. mutans*, with a minimum bactericidal concentration (MBC) of 1250 µg/mL and a minimum inhibitory concentration (MIC) of 625 µg/mL [15]. To further elucidate its mechanism of action, we examined morphological changes in bacterial cells after TC exposure. Treated *S. mutans* cells exhibited elongation and abnormal division, forming syncytium-like structures, cytoplasmic masses containing multiple nuclei without septation. This phenotype is characteristic of agents targeting bacterial cell walls, which typically lead to enlarged cells lacking septa [21].

Figure 2 highlights these morphological changes in the TC-treated groups. Notably, sub-MIC doses (≤625 µg/mL) led to increased bacterial density, suggesting enhanced bacterial survival and proliferation due to insufficient inhibition. These lower concentrations may trigger stress responses and survival pathways in *S. mutans*, potentially contributing to resistance development, an established risk associated with prolonged exposure to sub-MIC levels of antimicrobials. Therefore, using optimal, bactericidal concentrations is essential for long-term effectiveness.

**Figure 7:**
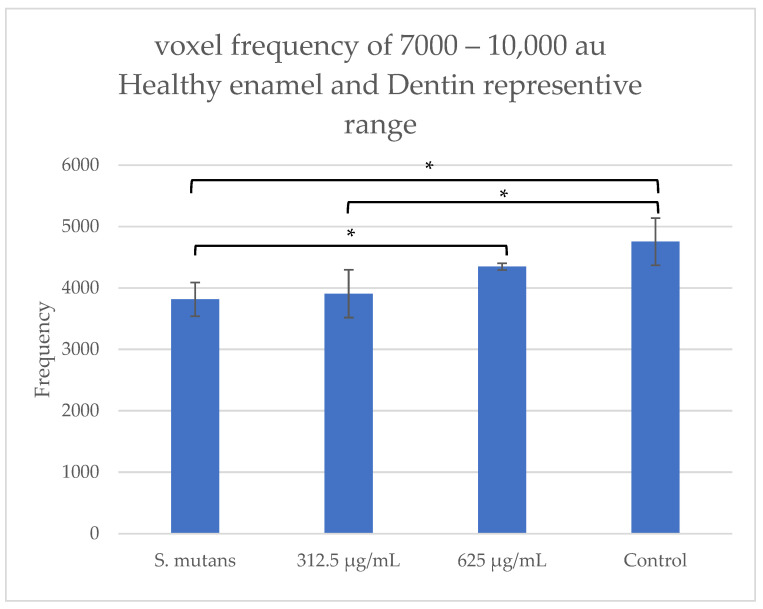
µCT analysis comparison of voxel frequency of healthy enamel and dentin representative ranges. Voxel frequency analysis of dental hard tissues at the 7000–10,000 interval showed a progressive increase in hard tissue density from the *S. mutans* group to the clear control. A significant difference was also observed between the *S. mutans* group and the 312.5 µg/mL TC group and between the 625 µg/mL TC group and the clear control group. * *p* < 0.05.

We observed a clear anti-cariogenic effect at TC concentrations ≥ 625 µg/mL, with no visible carious lesions forming, which correlates with the lowest concentration exhibiting antibiofilm activity [15]. Thereon, we proceeded to quantitatively evaluate the tissue loss, which resulted in the visible carious lesions in Figure 3, using Micro-Computed Tomography. We observed a similar pattern and correlation between the clinically visible carious lesions and the radiological demineralization with the clear control and the 625 µg/mL TC showing no radiological tissue loss, unlike the *S. mutans* group and the sub-MIC 312.5 µg/mL TC group, both in the coronal (frontal) plane and the 3D reconstruction (Figure 4). In the same light, the voxel frequency analysis showed that the clear control had the highest hard tissue density, followed by the 625 µg/mL TC, with the *S. mutans* group having the lowest hard tissue density. This further highlights the anti-cariogenic activity at the 625 µg/mL TC concentration (Figure 7).

As shown in Figure 5, these TC concentrations of ≥ 625 µg/mL maintained a stable pH of ~7 over the 5-day treatment period, remaining above the critical pH threshold (5.0–5.5) required for enamel demineralization and caries initiation. In contrast, lower concentrations and untreated controls experienced a steady decline in pH.

The ability of TC to prevent acidification is particularly important, considering that *S. mutans* continuously metabolizes dietary carbohydrates via glycolysis, producing acids that contribute to tooth demineralization when the surrounding pH drops below this critical level.

To confirm the necessity of bacterial involvement in caries formation, we replicated the three other key factors described in the modified Keyes diagram (tooth, sugar, and time) without the presence of bacteria. As shown in the negative control (Figure 3G), no caries developed, emphasizing the indispensable role of *S. mutans* in the caries process.

The in vitro murine jaw model [20] offers a rapid and ethically favorable alternative to in vivo experiments. Extracted hemi-mandibles from healthy, euthanized mice were used to control variables such as age, genetics, diet, and initial dental condition. Notably, murine molars share anatomical similarities with human molars, including fissures and grooves, making them a suitable model. Mandibles were chosen over maxillae for their greater reproducibility. Having said that, this model has some limitations, such as a lack of multispecies microbiota, clearance dynamics, salivary flow, and immune response.

We employed stereomicroscopy to evaluate carious lesions. This method provides a three-dimensional perspective, enhancing depth perception and allowing for more accurate identification of lesion presence or absence [22,23]. Post-treatment, a dose-dependent discoloration of the teeth was observed. This may be attributed to the high oxidative reactivity of TC, particularly its unsaturated aldehyde group, which can form stable bonds with surface proteins. While this staining could be an artifact of continuous exposure, unlike clinical scenarios, it warrants further investigation [24].

TC, an α,β-unsaturated aromatic aldehyde, offers several advantages: it is relatively inexpensive, widely available in nature (fruits, seeds, vegetables), exhibits low cytotoxicity, and shows high biochemical specificity. Importantly, due to its unique structure, TC is less prone to inducing bacterial resistance. However, its inherent instability when exposed to air, due to oxidation to cinnamic acid, may limit its shelf life or require formulation stabilization. To counter this instability, the use of a Schiff base form of TC may improve stability and possibly effectiveness.

As shown in Figure 6, post-treatment CFU counts mirrored the pH and lesion formation results. TC concentrations ≥625 µg/mL resulted in no detectable colonies, while lower concentrations showed bacterial growth. Notably, the 156.25 µg/mL group had higher CFU counts than the 312.5 µg/mL group, reflecting its reduced antibiofilm efficacy compared to the 312.5 µg/mL group.

Despite advances in understanding oral diseases, the global burden remains high. More than 800 million people are affected, indicating the limitations of current intervention strategies, such as the “drill and fill” approach (Cascada Declaration) [25]. Shifting toward primary prevention is crucial for reducing incidence and improving access to dental care.

Resistance to traditional antimicrobial agents is rising, further complicating treatment. A pivotal discovery in bacterial RNA regulation revealed a fluoride exporter and associated gene mutations in fluoride-resistant strains [12,26]. This highlights the urgent need to explore new therapeutic strategies.

Combining TC with other antibacterial approaches, such as blue light therapy [17], phage therapy [18], or cannabinoids [10,19], may enhance its anti-cariogenic efficacy through additive or synergistic effects.

## 4. Materials and Methods

TC (trans-cinnamaldehyde 99%, Sigma-Aldrich, Jerusalem, Israel)) was purchased and diluted progressively to a concentration range of 156.25–5000 µg/mL in dimethyl sulfoxide (DMSO) 0.03–1%, an organic solvent.

Our previous data insinuate that the most effective sub-MBC of TC is 625 μg/mL [15].

### 4.1. Cytotoxicity Assay

Cell toxicity was performed by seeding 60,000 human-immortalized keratinocyte (HaCaT) cells in 190 µL Dulbecco’s Modified Eagle’s Medium (DMEM) supplemented with 10% fetal calf serum (FCS) in a 96-well flat-bottom plate and incubating in a humidified incubator at 37 °C in the presence of 5% CO_2_. When the HaCaT cells were confluent and had attached to the bottom of the well, 10 µL of our different TC concentrations was added overnight. The following 100 µL per well were added to 100 µL of XTT (XTT-based colorimetric kit, Biological Industries Israel, Beit Haemek, Israel) and incubated for 2 h. The density emitted per well was analyzed at 450 nm and 650 nm using the M200 Tecan plate reader.

### 4.2. High-Resolution Scanning Electron Microscopy (HR-SEM)

Biofilms were allowed to form on sterile hydroxyapatite discs in the absence or presence of various concentrations of TC. After a 24 h incubation, the hydroxyapatite discs were rinsed with DDW (double-distilled water) and fixed in 4% glutaraldehyde in DDW for 40 min. The hydroxyapatite discs were washed again with DDW and allowed to dry at room temperature. The hydroxyapatite discs were then mounted on a metal stub and sputter-coated with iridium and visualized by a high-resolution scanning electron microscope (Magellan XHR 400L, FEI Company, Hillsboro, OR, USA). Three specimens from each treatment group were prepared and examined under SEM to evaluate the effect of TC on biofilm formation using sub-MBC concentrations.

### 4.3. Mouse Jaw In Vitro Caries Model

**Figure 8:**
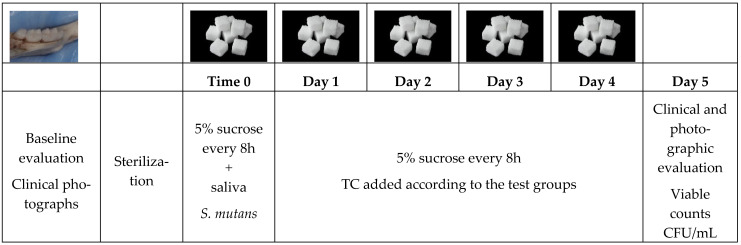
Schematic representation of our in vitro mouse experiment. All jaws were photographed prior to exposure to the caries-promoting environment using an SMZ25 stereomicroscope (Nikon, Tokyo, Japan). Then, all jaws were autoclaved. At time 0, jaws were subjected to saliva, bacterial infection by *S. mutans UA159*, and 5% sucrose growth media. Growth media was replaced every 8 h (4 in each group). After 5 days, microbiological samples were collected using a paper point.

Preparation of sacrificed 7-week-old BALB/C female mice hemi-mandibles was obtained from previously euthanized healthy mice. Simulate the oral cavity conditions by subjecting the samples to sterilized saliva once, followed by preparing a caries-promoting environment by adding bacterial suspension once as well (to create a biofilm) and BHI medium with 5% sucrose. *S. mutans* (OD600 nm = 0.1) were treated with different concentrations of TC and incubated at 37 °C for 24 h using the mouse jaw in vitro caries model. The media was changed every 8 h (3 times a day; consistent with the average number of times a person eats) as well as the administration of the TC concentrations for 5 days (Figure 8). On the 5th day, a clinical and photographic evaluation was conducted with the Nikon SMZ25 stereoscope (4 jaw explants in each group).

### 4.4. Micro-Computed Tomography (μCT) Evaluation of Jaw Explants

Jaws were scanned using an Ultra High Resolution Micro-CT System (μCT^UHR^, Milabs, Houten, The Netherlands) with an energy of 50 kV, a tube current of 240 µA, an exposure time of 75 ms, and a reconstructed voxel size of 40 µm. Total crown cubic volume (mm^3^) was determined by the marked rectangle over all slices in which crown enamel was analyzed quantitatively in three dimensions for mineral tissue loss. The sample scans were aligned using the method provided by Goldman et al. [27].

The analysis of the reconstructed images was conducted using Imalytics Preclinical software 3.1.1.8. In each scan, the region of interest (ROI) was defined as a box measuring 1.5 × 0.8 × 1 mm and was positioned at the cemento-enamel junction of the first molar. Twenty selected voxel-intensity frequency ranges (arbitrary units, a.u.) between 500 and 10,000 were obtained from each ROI. In a preliminary examination, an ROI within the control group containing only healthy enamel and healthy dentin was identified. All sample volumes consisting exclusively of healthy enamel and dentin remained within a density range of 7000–10,000 arbitrary units. The mean frequency within the representative healthy enamel and dentin range (7000–10,000 a.u.) was compared between groups.

### 4.5. Post-Treatment Viable Count Evaluation

Sterile paper points were used to collect biofilm from the tooth surface of the molars after the 5th day. The paper points were placed in an Eppendorf tube with 100 µL PBS and sonicated for 10 min to disrupt the biofilm (Bandelin sonopuls HD 2200, Berlin, Germany). Then, the tube was vortexed, and progressive dilutions were carried out and plated on BHI agar plates (Difco, Detroit, MI, USA) for live bacterial count evaluation (CFU/mL).

### 4.6. pH Measurements

At various time points during the experiment (every 8 h), the pH of the samples was measured using pH indicator strips (MColorpHast, Merck KGaA, Darmstadt, Germany).

### 4.7. Statistical Analysis

The results were analyzed using one-way ANOVA followed by the Tukey–Kramer post hoc test. All *p*-values < 0.05 were considered statistically significant.

## 5. Conclusions

This study demonstrates that trans-cinnamaldehyde exhibits dose-dependent anti-cariogenic effects against *S. mutans* in a validated mouse jaw explant model. A concentration of 625 µg/mL was identified as the lowest effective and non-toxic dose, capable of maintaining neutral pH, inhibiting bacterial growth, and preventing caries formation. These findings underscore TC’s potential as a natural and effective agent for caries prevention while also contributing to the development of ethically sound in vitro models and alternatives to conventional antimicrobial therapies. The antibacterial properties of TC may contribute to the development of innovative formulations for both domestic and therapeutic use, which could help inhibit the growth of oral bacteria and thereby assist in the prevention of dental caries.

## Data Availability

The original contributions presented in this study are included in the article. Further inquiries can be directed to the corresponding author.

## Abbreviations

The following abbreviations are used in this manuscript:

TC	Trans-cinnamaldehyde
*S. mutans*	*Streptococcus mutans*
MBC	Minimum bactericidal concentration
DALYs	Disability-adjusted life years
DMEM	Dulbecco’s modified Eagle’s medium
DMSO	Dimethyl sulfoxide
DDW	Double-distilled water
MIC	Minimum inhibitory concentration
